# Heterogeneity Influence of Financial Digitalization and Inclusion on Employees’ Psychological States

**DOI:** 10.3390/bs13030263

**Published:** 2023-03-16

**Authors:** Yue Lu, Zuoqian Zhang, Siying Yang

**Affiliations:** 1 School of Business and Management, Jilin University, Changchun 130012, China; 2 School of Business, Qingdao University, Qingdao 266075, China; 3 School of Economics, Liaoning University, Shenyang 110136, China

**Keywords:** mental health, digital inclusive finance, income

## Abstract

Digital inclusive finance (DIF) has the power to spawn a new system of Internet finance and realize financial inclusion. However, the role of DIF in improving the health status of individuals is largely unknown. This study aims to demonstrate whether and how the development of DIF impacts the mental health of Chinese employees. This paper performs an empirical study based on the city-level data of the digital inclusive financial index with the China family panel studies. Ordinary least squares (OLS), probit models and mediation techniques are employed with appropriate instruments to alleviate endogeneity concerns. The results show that DIF can help employees improve their mental health. The results were robust to a variety of checks. Moreover, increasing income is the main pathway in which DIF improves individual mental health. Finally, it also reveals the heterogeneous effects of DIF on individual mental health. That is, the use depth of DIF has a significant positive effect on mental health status, but not on other sub-indicators, such as coverage breadth and degree of digital service provision; on those vulnerable groups containing females and employees with low education, its decisive role is larger than their counterparts who are males and have high levels of education. These results highlight the vital role of DIF in improving the mental health status of individuals. Consequently, there is a need to strengthen the construction of financial infrastructure and achieve a deeper integration of the financial system with digital technologies.

## 1. Introduction

With the extremely rapid development of China’s economy, and the continuous improvement of employees’ living conditions, most people’s pursuit of health gradually shifts from physical to mental health. Mental health and greater well-being have also become an important part of the United Nations Sustainable Development Goals. However, at this stage, the state of the mental health of Chinese employees is not looking optimistic. A recent paper indicates that 4.3 million Chinese adults have a serious mental illness [1]. Other research also points out that mental illness caused by depressive symptoms has evolved into the leading cause of suicide [2]. Against this background, the Chinese government decided to raise the issue of mental health to a national strategic level [3]. Social stress is an important factor affecting mental health, especially financial stress [4]. DIF, as one of the forms of the digital economy, has strong resource availability and more efficient resource allocation [5]. It appears to be an important channel for the employee to mitigate income risks, and thus, probably improve their mental health. However, is this situation accurate?

To sum up, the extant literature reveals the determinants of mental health mainly from four aspects: the first is financial factors, including relative economic status and personal income [6,7]; the second is political factors, mainly in terms of government financial assistance and corruption [8,9]; the third is socio-demographic characteristics, including age, gender, marriage, education level and occupations [10,11]; the fourth is external environmental factors, such as air pollution and natural disasters [12,13]. While the above factors, including individual and household endowments, institutional arrangements, and the external environment, are important, less attention has been paid to the consequences of applying new technologies.

As an important part of the modern financial system, inclusive finance was formally introduced in 2005 to balance efficiency and equity, facilitate access to financial services for low-income groups excluded from the formal financial system, and promote inclusive economic growth [14]. In recent years, DIF has penetrated the daily lives of Chinese workers [15], such as mobile payment, online loans, Internet insurance, smart Internet finance, and other new financial products. By the end of June 2021, the number of users that opened mobile payments reached 872 million, accounting for 86.3 percentage points of all netizens, and the transactions were more than CNY 400 trillion.

Several recent studies of the DIF are focused on evaluating macroeconomic effects, especially regarding how to promote green innovation and achieve the integration of rural three-industry [16,17]. From the perspective of micro-level effects, other studies also show the positive effect of DIF on alleviating poverty [14,18], household entrepreneurial decisions [19], consumption structure [20], investment portfolio efficiency [21], and debt ratio [22]. However, there has been insufficient research to discuss the role of DIF on employees’ mental health. To our knowledge, only three relevant studies investigate the effect of inclusive finance on individual’s psychological health, respectively [23,24]. These findings suggest that financial inclusion has an important impact on mental health. Still, the analytical sample is mainly from Africa and North America, and the selection of financial inclusion indicators is relatively homogeneous, which cannot reveal the new connotation of DIF.

Using a combination of China family panel studies (CFPS) data and the digital inclusive financial index, we empirically identify the effect of DIF on individuals’ mental health. Specifically, our study confirms that DIF in general significantly improves the mental health of individuals, and that this conclusion holds after employing alternative measurements and estimation methods. In the meantime, mechanism exploration indicates that increasing the employees’ income is the potential channel through which DIF promotes mental health. Heterogeneity analysis further shows that only the use depth of DIF has a significant positive effect; on those vulnerable groups, such as females and employees with low education, who may benefit more from the rapid development of DIF. Our results can offer policy implications to help governments facilitate the development of DIF in improving the mental health of employees in China.

The rest of the paper is organized as follows. The literature review section reviews and summarizes the relevant studies. The research methodology section presents the data sources, variable definitions, and model specification. The results section gives the results of the baseline estimation, instrumental variables, and robustness test. The further discussion section gives the effects of different dimensional sub-indicators of the digital inclusion finance on individual mental health and the heterogeneity effects of digital inclusion finance on different groups. The mechanism test section explores the possible mechanisms. Finally, conclusions and policy recommendations are presented.

## 2. Literature Review

Scholars have studied health issues at the individual, family, and societal levels. Among the studies at the individual level, all point to economic stress as an important source of increased psychological stress and the deterioration of mental health among workers [3], income deprivation leads to poorer self-rated health and mental health [25]. Buttrick et al. argue that economic inequality (wealth gap, unequal income distribution or income inequality, etc.) can bring about mental health problems, increasing mental illness and decreasing life satisfaction and well-being [26]. Among them, self-employed individuals also experience less negative emotions than employed individuals [27,28]. Mathieu et al. state that unemployment often brings a shock to an individual’s financial situation, which affects the individual’s mental health [29]. Purtle argues that stress caused by financial insecurity may increase the risk of depression and suicide [30]. Hä mmig et al. further noted that the physical and psychological health of workers in working conditions is significantly higher than average [31]. Thus, income may prevent psychological distress [32].

Financial stress is an important cause of increased psychological stress, and even deteriorating health [33,34,35]. In recent years, the development of digital inclusive finance in China has eased financial stress, and thus, improved mental health. For one, first, in terms of direct effects, digital inclusive finance can optimize the financial environment, reduce the degree of financial exclusion of micro individuals from formal financial institutions, and bring down the threshold and cost of formal finance. This not only reduces the income mobility constraint of workers, but also increases the participation of productive investment behaviors, such as entrepreneurship, through formal financing, which in turn increases income [22,36], achieves the optimal allocation of income and consumption over one’s life cycle, which increases consumption and improves workers’ mental health [37]. Second, the development of digital inclusive finance is conducive to creating a more equitable social environment and mitigating the welfare loss caused by financial exclusion. It reduces the cost of labor transfer and industrial upgrading, raises the status of human capital in income distribution, increases the share of labor income, reduces the gap between rich and poor caused by the unequal development opportunities and has a positive impact on psychological health [38].

It is worth noting that with the construction of the inclusive financial system, the positive role of digital inclusion in achieving social inclusion has become more evident. The positive effect of digital inclusive finance on the mental health of disadvantaged groups has become more obvious. It helps remove barriers to financial capital flows and creates an equitable financial environment, which enables disadvantaged groups to improve their social environment through access to high-quality financial services [39]. It helps disadvantaged groups to obtain equitable rights and improves workers’ self-esteem and self-motivation, thus improving their mental health [40].

The contributions of this paper are as follows. From a research perspective, few scholars have studied the economic effects of digital financial inclusion from a psychological health perspective, especially for developing countries, where people are under greater economic stress. For this reason, the impact of inclusive financial development on the mental health of Chinese residents is explored from a micro perspective, which helps in the understanding of the relationship between digital financial development and micro individual behavior. In terms of research methodology, the possible endogeneity between financial inclusion and mental health is often overlooked. Therefore, we examined the impact of digital financial inclusion on mental health using instrumental variable and lagged variables methods. In terms of research content, we have made a more comprehensive examination of the impact of digital inclusive finance and its sub-indicators on mental health and its mechanisms and heterogeneity. In conclusion, our study answers the question of whether the development of digital inclusive finance affects individual mental health and how digital inclusive finance affects individual mental health, which has important theoretical and practical implications. In addition, based on China, a typical developing country, its findings have important practical implications for the development of digital inclusive finance in developing countries.

## 3. Methodology

### 3.1. Data

The data we use include both macro and micro levels. Specifically, the micro-level data has stemmed from the CFPS, which is conducted by the Institute of Social Science Survey at Peking University. CFPS is a nationally representative, biennial longitudinal survey (the CFPS is a widely accessible data set. Specifically, it uses three stages of the stratified sampling method and the probability proportional to size sampling strategy to survey residents who have lived in 25 provinces in both rural and urban China) and the purpose of the project is to collect rich information at the individual, household, and community levels through face-to-face and telephone interviews to reflect China’s demographic and socioeconomic characteristics, thus providing a basis for relevant public policy formulation and academic research. To date, five waves of surveys have been carried out, namely in 2010, 2012, 2014, 2016, and 2018. Based on the research objectives, we selected only the 2014 wave for analysis (the 2014 CFPS data were specifically selected as the analytical sample, primarily to ensure the consistency of the measures. There is some variation in the question and response options regarding individual mental health in multiple waves of CFPS data. If a balanced panel or pooled cross-sectional data set is constructed using data from different years, it may cause measurement errors in mental health indicators to a large extent) and kept only individuals involved in non-agricultural employment activities (in the questionnaire, respondents’ job types were classified into four categories: farmers, private enterprises/self-employed entrepreneurs, agricultural workers, and off-farm workers. In this paper, only individuals engaged in non-agricultural employment activities were retained (i.e., occupations belong to the second and fourth categories)). After eliminating observations with missing information and obvious outliers in all variables, we finally obtained a valid data set consist of 10,193 individuals distributed across 112 cities in 24 provinces.

In terms of the macro-level data, it mainly comes from the DIF index in China released by the Institute of Digital Finance at Peking University (The index is derived from the research reports. Available online: https://idf.pku.edu.cn/attachments/d67f649195fd4a7ea8082d1324de7e78.pdf, accessed on 1 July 2016). To characterize the evolution of China’s digital finance system, the Institute of Digital Finance and the Ant Financial Services Group initiated a joint project in 2011. Specifically, these indexes consist of the aggregate DIF index and three secondary indicators, as well as multiple specific third-level indicators. It should be emphasized that the index of DIF is the most representative indicator to reveal financial inclusion in China, and has been widely adopted to demonstrate the economic and social effects of the digital economy [41,42]. Meanwhile, this data set embodies three levels of DIF index: province, municipality, and county. Our study employed the data at the municipal level for empirical analyses. As a robustness check, we also use the provincial aggregated index to match micro data.

### 3.2. Variables

#### 3.2.1. Dependent Variables

Our dependent variable of primary interest is mental health. Following the practice of mainstream studies on psychological health [34], we create multiple ordinal variables based on the six questions: ‘During the past month, about how often did you feel: (1) Nothing could cheer you up? (2) Nervous? (3) Restless and cannot remain calm? (4) Hopeless about the future? (5) Everything is difficult? (6) Meaningless?’ For each of the six questions, the respondent selects one of five options: 1 = almost every day, 2 = often, 3 = half of the time, 4 = sometimes, 5 = never. In this study, a greater value denotes an improved mental state.

#### 3.2.2. Independent Variables

The key independent variable was the development degree of DIF. We used the DIF index at the municipal level and matched it to the city where the respondent was located. It is noteworthy that the regional DIF index was constructed based on the consumer big data of Alipay. At the same time, to examine the heterogeneous effects, we further chose three sub-dimensions of the DIF: coverage breadth, the use depth, and degree of digital service provision. More specifically, regarding the coverage breadth, it involves one secondary indicator, which is account coverage, which contains several third-level indicators: the amount of Alipay accounts per 10,000 people, the mean value of bound debit or credit cards per Alipay account, and the percent of Alipay users. From the perspective of use depth, it covers six secondary indicators: payment, monetary funds, loans, investment, insurance, and credit investigation, similarly comprising multiple third-level indicators such as per capita payment. Finally, in terms of the degree of digital service provision, it also reflects four secondary indicators: affordability, mobilization, facilitation, and credit, as well as includes ten third-level indicators (e.g., the percent of payment frequency with mobile and the average loan interest rate of individuals).

#### 3.2.3. Control Variables

Referring to the practices in mainstream research on mental health in China [43,44,45,46], we included a battery of control variables to reflect individual, household, and city level characteristics. First, individual-level factors included in the empirical design are age, male, marital status, *Hu-Kou*, and years of education. *Marital status* is a dichotomous variable that equals 1 if the respondent is married and 0 if otherwise. *Hu-Kou* is coded 1 if the respondent has urban. Next, we included the number of household members, the elderly dependency ratio (aged 60 years and older), the child dependency ratio (under 16 years of age), the household’s entrepreneurship, and the natural logarithm of the total liabilities to consider household-level factors. Furthermore, to reveal the effect of the city-level characteristics, we also controled *GDP*, *population size*, and *financial development level* (we obtained this information from the 2014 regional statistical yearbooks. Considering the potential non-normality of GDP per capita and population size, these variables were adjusted to natural logarithms in the regression analyses). Table 1 reports the descriptive statistics for each variable in the analysis.

As seen in Table 1, the respondents’ mental health is good. On average, the total index of DIF is 1.375, with a minimum value of 0.932 and a maximum value of 1.893, suggesting that the development of DIF is not balanced among cities, which enables us to evaluate its effect. Turning our attention to other individual and household characteristics, the mean age of the respondents was 39 years old, including 58.5% males, 82.7% married with a spouse, and 41.8% urban employees. The average years of schooling was 9.792, suggesting that many of them have education attainment beyond junior high school. The number of family members was 4.24. Regarding the dependency ratio, the percentages of respondents with any elderly population or children were 11.2% and 14.5%, respectively, within a reasonable range. The likelihood of a household starting a business was 16.3%. Additionally, household information also reports that the logarithm of the total debts is 3.73, which implies that some families have debt burdens. In terms of city-level characteristics, the means in the GDP per capita, population size, and financial development level are 10.80, 6.28, and 1.03, respectively, which are also realistic.

The Pearson correlation coefficients between the DIF index and individual mental health indicators were also calculated prior to the regression analysis. Specifically, since the former is a city-level index and the latter is an individual-level variable, we used the latter to calculate the city-level mean of each mental health index, and finally, calculated the correlation coefficients based on the data of 112 prefecture-level cities (by the end of 2020, the number of prefecture-level or above cities in China was 293. The CFPS survey covers only half of the cities, but the project follows the principle of randomness in the sampling, so the data can be considered nationally representative). Table 2 displays the simple correlation matrix of all the key variables. The results uncover at least two important messages. First, the six mental health indicators are highly correlated. Although there are differences in what they reflect, their correlation coefficients are above 0.7 and significant at the 1% statistical level. Second, the DIF index was positively correlated with each individual mental health indicator and was statistically significant, at least at the 10% level. Based on the above results, although we can intuitively determine that DIF helps improve individuals’ mental health, more rigorous causal inferences depend on further regression analysis.

### 3.3. Model Specification

Regarding the estimation strategy, since an individual’s mental health is measured by ordered variables, the ordered probit model should strictly be adopted for estimation. However, previous studies have shown that the marginal effects of estimated parameters from non-linear models are not that different from the coefficients from linear estimations [47,48]. Meanwhile, scholars also posit that compared to non-linear models (ordered probit) [49,50], the OLS regression is more intuitive and interpretable by a wide range of audiences. Therefore, to quantitatively assess the role of the DIF in improving an individual’s health status, we estimate the following equation using the cross sectional data:

(1) $$Y_{i,j,k}=\delta_{0}+\delta_{1}DIF_{i,j,k}+\delta_{2}X_{i,j,k}+\delta_{3}Z_{j,k}+\varepsilon_{i,j,k}$$

Equation (1) presents our baseline model. Where the subscripts i, j, and k represent the individual/household, city, and province, respectively. The dependent variable $Y_{i,j,k}$ indicates individual i’s mental health in city j in province k. $DIF_{i,j,k}$ measures the development degree of DIF in city j of province k. $X_{i,j,k}$ is a matrix of controls at the individual and household levels. $Z_{j,k}$ represents a vector of city-level characteristics. $\varepsilon_{i,j,k}$ is a random error term. We employ the 1-period lagged independent variables and city-level controls to alleviate the issues of reverse causality. Meanwhile, $\delta_{1}$ is the estimated coefficient of interest, which explores the nexus between DIF and mental health, and we expect $\delta_{1}$ to be positive. Finally, our study utilizes robust standard errors at the municipal level to allow for heteroscedasticity in the estimations.

## 4. Results

### 4.1. Baseline Estimates

Table 3 presents the OLS estimates of the effect of DIF on individuals’ mental health. As shown from the results in the table, six dimensions of mental health indicators showed a significant and positive effect at the 1% level, which suggests that the thriving development of DIF can substantially improve the mental health of individuals. This finding of the positive effect echoes the conclusion of Li et al. and Zhang et al. [41,51]. They use income and consumption as outcome variables, demonstrating that the development of Fintech (defining the DIF index as a proxy) is positively associated with household income and consumption. This paper also attempts to use stepwise regressions to confirm the robustness of the results, that is, adding individual-, household-, and city-level control variables in turn. These findings indicate that the coefficients on DIF are positive and significant. We do not provide the series of estimation results in the manuscript due to space limitations.

Concerning the control variables, our results are generally consistent with the previous literature [45,46]. For example, it was found that male and married respondents can report higher levels of mental state than female ones. Additionally, we found that the household’s debt and financial development level can an deteriorate individual’s mental health.

### 4.2. Endogeneity Analyses

Although the regression results suggest that the development of DIF can encourage an individual’s mental health, endogeneity issues may arise in the model specification due to omitted variables or reverse causation, thus leading to an array of biased estimates in our empirical analysis. Specifically, although we included individual-, family-, and city-level controls in our baseline model, we were generally unable to account for all the factors (e.g., personality, family background, and working environment) that influence individuals’ mental health. On the other hand, mental health status may also impact individuals’ migration decisions or financial product use behaviors, which may affect digital inclusion in their cities. Consequently, our study will re-estimate the above regression results using the lagged variable method and the instrumental variable (IV) strategy, respectively, in order to alleviate possible endogeneity problems.

Prior research had frequently adopted endogenous independent variable lag term as instruments to solve the issue of endogeneity [34]. Given the DIF index is published from 2011 and the core independent variables in the baseline model are 2013 data, the maximum lagged period that can be chosen for this study is two periods, we re-estimate the above econometric model in conjunction with data availability, using the DIF index with one and two lags as the key independent variables, respectively. Panels A and B in Table 4 report the corresponding estimation results, and we can see that the coefficient of the DIF index remains significantly positive with longer lags. Hence, the preliminary conclusion that there is a positive link between the development of DIF and the mental health of individuals is credible.

In addition, our study uses the IV method to address the endogeneity problem further and to strive to identify the net effect of the DIF on mental health. Following previous studies [52,53], we chose the distance with the specific provincial capital as an IV to capture the exogenous variation in DIF, and adopted a two-stage least square (2SLS) approach to re-estimate our econometric model. Generally speaking, the distance between an individual’s city and the provincial capital is a variable that involves the relative distance based on GIS measurements. From a theoretical point of view, geographic distance has a strong correlation with economic development and digital infrastructure construction. Provincial capitals are usually a province’s political and economic centers, so they can be considered as the most mature areas for the digital finance system. The distance from the provincial capitals largely indicates the development degree of digital financial inclusion. At the same time, we expect that it is unlikely that it will impact the employees’ mental health. Hence, our study argues that the instrument would meet both the relevance and exogeneity assumptions of the IV method.

Table 5 reports the results of the IV analysis. As shown in Column (1), the first-stage estimates demonstrate that the coefficient of the IV is negative and significant at 1% level. Columns (2)–(7) report the set of second-stage results. Using distance from the provincial capital as IV, we continue to find that the rapid development of DIF remains to have a significant and positive effect, which is consistent with our findings in the baseline estimates. Finally, the results based on the weak IV test show that the F statistic is higher than the corresponding critical value with the tolerance of 10% offered by Stock and Yogo [54], which means that there is no weak IV in our model specification. In a similar vein, according to the identifiable test results of IV, the LM statistic also rejects the null hypothesis that the equation is under-identified at the 1% level. We thus conclude that the estimated IV results are effective.

### 4.3. Robustness Checks

To validate whether our baseline estimates are biased, we also performed an array of robustness checks by using alternative measurements and other estimation approaches.

First, we chose the provincial aggregated DIF index in our study to reflect the rapid development of digital inclusive finance. Table 6 reports the coefficients. The findings are similar to our main OLS results in Table 3. Second, given that our outcome variables are subjective measures, we reset the outcome variables to dichotomous variables to address measurement errors. Specifically, in accordance with the mean values of the six mental health indicators, we defined six dummy variables separately. If the respondent’s mental health is above the median value, then the value is assigned to 1. Otherwise, it is 0. We next employ the probit model to re-estimate the marginal effects of DIF on mental health. Panel A in Table 7 shows that the development of DIF substantially promotes individual mental health. Finally, the OLS approach will likely lead to biased estimates since the outcome variables are ordered categories variables. Therefore, we adopted a maximum likelihood estimation, preferred by most scholars [50], and performed a regression analysis by constructing an ordered probit model. The estimation results are shown in Panel B, and these coefficients of interest are largely consistent with our baseline estimates. We continue to conclude that the DIF has a positive effect on the mental health of Chinese employees.

## 5. Discussion

The analysis in the above sections has demonstrated that the development of DIF has significantly improved employees’ mental health and confirmed the robustness of this preliminary conclusion. Next, we will further discuss the potential mechanism through how DIF impacts mental health, as well as the disparities in the effects of the sub-dimensions of DIF and different groups of respondent.

### 5.1. Mechanism Exploration

In the baseline estimates, we first conclude that the connection between DIF and mental health is positive and significant. Next, referring to the practice of Preacher and Hayes [55], and by estimating Equations (2) and (3), we further examine whether the mediation effect is statistically significant.

(2) $$Mechanism_{i,j,k}=\theta \alpha_{0}+\theta_{1}DIF_{i,j,k}+\theta_{2}X_{i,j,k}+\theta_{3}Z_{j,k}+\varepsilon_{i,j,k}$$

(3) $$Mental\;health_{i,j,k}=\gamma_{0}+\gamma_{1}DIF_{i,j,k}+\gamma_{2}Mechanism_{i,j,k}+\gamma_{3}X_{i,j,k}+\gamma_{3}Z_{j,k}+\varepsilon_{i,j,k}$$

where $Mechanism_{i,j,k}$ represents the mediator, we employ net income per capita as proxy. Other variables are defined as in Equation (1). Specifically, the first step is to conduct a regression analysis on Equation (1). $\delta_{1}$ is the estimated coefficient that reveal the total effect of DIF on mental health. The second step is to perform a regression analysis on Equation (2). Coefficient $\theta_{1}$ denotes the extent to which DIF affects employees’ net income (if significant, it implies that the DIF has explained the variation of the possible mediator). The third step is to conduct a regression analysis on Equation (2), and the $\gamma_{2}$ measures the impact of the mediator on mental health after controlling the DIF index. If $\gamma_{1}$ and $\gamma_{2}$ are significant and have the signs as expected, the magnitude of the coefficient of $\gamma_{1}$ is lower than $\delta_{1}$, which suggests that there is a mediating effect to some extent.

The results of the mechanism exploration are presented in Table 8. We first adopted an OLS model to estimate the impact of DIF on personal net income in Column (1). As shown in the rest of the columns, we also found that the DIF remains significantly and positively associated with individual mental health even when we contain a mediator in the model specification. Therefore, we argue that digital inclusive finance can substantially improve mental health by increasing net income. This suggests that digital inclusive finance facilitates a fair financial environment, lowers the threshold and cost of borrowing, helps people cope with income liquidity risks, and alleviates the financial stress caused by shortage of funds [38]. Higher income increases people’s confidence in their future lives, which improves mental health.

### 5.2. Heterogeneity Analyses

One more interesting problem deserving investigation is whether the nexus between DIF and an individual’s mental health differ across several important aspects, such as the sub-indicators of DIF, education level, and gender.

#### 5.2.1. Heterogeneity in the Sub-Indicators of DIF

Digital inclusive finance is a multidimensional concept. For instance, it can be reflected in the improvement of efficiency and cost of financial services, as well as in the deepening of Internet financial services and the increasing number of transaction accounts. Hence, this paper not only investigated the effects of the total index of DIF on individual mental health but also adopted the second-level indicators in the empirical analyses.

Specifically, the sub-indicators are coverage breadth, use depth, and degree of digital service provision. Panel A in Table 9 reports the estimation results based on the sub-indices of DIF. We find that only the coefficient of the use depth is significantly positive, while the coefficients of the coverage breadth and degree of digital service provision both show negative signs. This finding suggests that the system of China’s digital inclusive finance may not be able to satisfy employees’ real needs effectively. Currently, the shortcomings of inclusive digital finance, such as smaller coverage, higher service threshold, and lower operational efficiency, seriously limit its role in improving individual mental health.

#### 5.2.2. Heterogeneous Effects by Education Level and Gender

Because the preceding sections discuss only a homogeneous effect of DIF on employees’ mental health, we also test for the presence of heterogeneity by estimating the impact by education level and gender. In a word, our study includes the interaction term of the DIF index and the binary variable for the education level and gender, respectively. On the one hand, according to the difference in the education level of the respondents, the value of high school or above is 1; otherwise, it is 0. On the other hand, according to the difference in gender, the value of a male sample is 1, and the female is 0. As Panels B and C in Table 9 show, we confirm that the coefficients of the interaction term of DIF with human capital and gender are significantly negative, indicating that the mental health of those respondents who are male or more educated is less positively affected by digital inclusion compared to those who are less educated and are female. This suggests a clear positive role of digital inclusive finance in achieving social inclusion [39]. Digital inclusive finance creates an equitable financial environment, which enables more disadvantaged groups (women and people with low education levels) to achieve a higher income through high-quality financial services, increasing people’s self-esteem and self-motivation, which improves mental health.

## 6. Conclusions

Currently, the development of digital inclusive finance has been thriving because of the Internet and cloud computing. Using a combination of CFPS data and the digital inclusive financial index, this study conducted empirical analyses on the effects of DIF on employees’ mental health in China, and examined its underlying channel and heterogeneous effects. Specifically, the estimation results suggest that DIF can help employees significantly improve their mental health, and that this conclusion holds after correcting the endogenous biases and performing a series of robust checks. The mediation model indicates that net income per capita is a mediator in the connection between DIF and mental health, which implies the positive effects of DIF on individual mental health mainly through mitigating income risks. Furthermore, we demonstrated the heterogeneous effects. Among the sub-indicators, the use depth of DIF had a significant beneficial role, but not the coverage breadth and degree of digital service provision; on those who are female and the employees with low education, its positive impact is larger than on their counterparts who have high levels of education and are male.

Our findings have important policy implications. First, people are currently facing greater economic pressure, the physical and mental health, especially their psychological health, should receive attention. Therefore, along with technological progress, we should pay attention to its impact on workers’ mental health. Next, countries around the world should pay attention to the development of digital inclusive finance, continue implementing a long-range plan for the development of DIF, promoting the construction of financial infrastructure and achieving the in-depth integration of traditional finance and digital technology. For example, a well-functioning credit investigation platform should be constructed to ease the information asymmetry between supply sides and users. Finally, digital inclusive finance plays a critical role in fostering the mental health of vulnerable groups, but these people are the weak link in digital inclusive finance development due to their shortcomings in economic status and behavior habits. Therefore, some projects aimed at benefiting employees (e.g., bringing smartphones to rural areas) should be accelerated.

Obviously, this study is not without limitations. First, due to microdata limitations, this paper uses cross-sectional data for 2014. Compared with panel data, the empirical results of cross-sectional data may not be very robust, making the empirical results inevitably regrettable. Additionally, it cannot account for the time effect of digital financial inclusion on individuals’ mental health. Similarly, due to data availability, this paper measures the development of digital inclusive finance at the provincial and municipal levels, which cannot accurately reveal the extent to which individuals benefit from using digital inclusive finance tools. Second, although we have explored the mechanisms underlying the effects of digital inclusion on individual mental health, we have not examined the conditions on which they rely. Future research could further explore this at the micro-individual level, thereby increasing the factor endowment of Chinese employees. Future research could consider using a more comprehensive data set for this study. Third, this study focuses on the impact of the development of digital inclusion finance on mental health. The development of digital inclusive finance may affect behavioral health, which has broader policy implications.

## Figures and Tables

**Table 1:** Descriptive statistics of main variables.

Variables	Obs.	Mean	Std. dev	Min	Max
Nothing could cheer you up	10,193	4.259	0.869	1	5
Nervous	10,193	4.415	0.836	1	5
Restless and cannot remain calm	10,193	4.548	0.755	1	5
Hopeless	10,193	4.735	0.639	1	5
Everything is difficult	10,193	4.545	0.747	1	5
Meaningless	10,193	4.763	0.600	1	5
Digital financial inclusion	10,193	1.375	0.248	0.932	1.893
Age	10,193	39.75	12.52	16	83
Male	10,193	0.585	0.493	0	1
Marital status	10,193	0.827	0.378	0	1
Urban	10,193	0.418	0.493	0	1
Years of education	10,193	9.792	3.904	0	22
Number of household members	10,193	4.244	1.835	1	17
Elderly dependency ratio	10,193	0.112	0.199	0	1
Child dependency ratio	10,193	0.145	0.160	0	0.714
Household entrepreneurship	10,193	0.163	0.369	0	1
Total household liabilities (*CNY*, Log)	10,193	3.731	5.236	0	12.95
GDP per capita (*CNY*, Log)	10,193	10.80	0.787	9.037	12.58
Population size (10 thousand, Log)	10,193	6.277	0.645	4.680	8.119
Financial development level	10,193	1.026	0.583	0.184	2.948

**Table 2:** Correlation matrix.

	Digital Inclusive Finance	Nothing Could Cheer You Up	Nervous	Restless and Cannot Remain Calm	Hopeless	Everything Is Difficult	Meaningless
Digital inclusive finance	1.000						
Nothing could cheer you up	0.107 *	1.000					
Nervous	0.205 **	0.797 ***	1.000				
Restless and cannot remain calm	0.215 **	0.786 ***	0.778 ***	1.000			
Hopeless	0.204 **	0.787 ***	0.757 ***	0.791 ***	1.000		
Everything is difficult	0.298 ***	0.765 ***	0.739 ***	0.802 ***	0.772 ***	1.000	
Meaningless	0.248 ***	0.758 ***	0.727 ***	0.718 ***	0.870 ***	0.804 ***	1.000

Note: * *p* < 0.1; ** *p* < 0.05; *** *p* < 0.01.

**Table 3:** OLS estimates for the impact of DIF on individual’s mental health.

	(1)	(2)	(3)	(4)	(5)	(6)
	Nothing Could Cheer You up	Nervous	Restless and Cannot Remain Calm	Hopeless	Everything Is Difficult	Meaningless
Digital inclusive finance	0.524 ***	0.513 ***	0.278 ***	0.451 ***	0.474 ***	0.405 ***
	(0.105)	(0.104)	(0.094)	(0.077)	(0.090)	(0.076)
Age	−0.006	−0.013 ***	−0.004	−0.010 ***	−0.011 ***	−0.012 ***
	(0.005)	(0.005)	(0.004)	(0.004)	(0.004)	(0.003)
Age squared	0.011 **	0.021 ***	0.006	0.012 ***	0.015 ***	0.013 ***
	(0.006)	(0.005)	(0.005)	(0.004)	(0.005)	(0.004)
Male	0.127 ***	0.102 ***	0.110 ***	0.077 ***	0.048 ***	0.106 ***
	(0.018)	(0.017)	(0.016)	(0.013)	(0.015)	(0.013)
Marital status	0.073 **	0.052 *	0.052 **	0.100 ***	0.102 ***	0.076 ***
	(0.029)	(0.028)	(0.025)	(0.023)	(0.026)	(0.021)
Urban *Hu-Kou*	−0.058 ***	−0.058 ***	−0.052 ***	−0.050 ***	−0.036 **	−0.018
	(0.200)	(0.019)	(0.017)	(0.015)	(0.017)	(0.014)
Years of education	0.007 **	0.002	0.014 ***	0.010 ***	0.012 ***	0.011 ***
	(0.003)	(0.003)	(0.002)	(0.002)	(0.002)	(0.002)
Number of household members	0.009 *	−0.001	0.003	0.007 *	−0.002	0.004
	(0.005)	(0.005)	(0.005)	(0.004)	(0.004)	(0.004)
Elderly dependency ratio	−0.028	−0.060	−0.001	−0.071 *	−0.062	−0.069 *
	(0.049)	(0.047)	(0.045)	(0.039)	(0.045)	(0.038)
Child dependency ratio	−0.117 *	−0.014	−0.029	−0.019	0.050	0.021
	(0.061)	(0.059)	(0.053)	(0.044)	(0.052)	(0.042)
Household entrepreneurship	−0.024	0.002	−0.009	0.042 **	0.002	0.030*
	(0.024)	(0.022)	(0.021)	(0.016)	(0.020)	(0.016)
Total household liabilities	−0.008 ***	−0.009 ***	−0.009 ***	−0.006 ***	−0.011 ***	−0.006 ***
	(0.002)	(0.002)	(0.002)	(0.001)	(0.002)	(0.001)
GDP per capita	−0.069 ***	−0.081 ***	−0.022	−0.076 ***	−0.047 **	−0.056 ***
	(0.025)	(0.024)	(0.022)	(0.018)	(0.021)	(0.017)
Population size	−0.015	0.022	0.013	0.009	−0.001	0.007
	(0.017)	(0.016)	(0.015)	(0.012)	(0.015)	(0.011)
Financial development level	−0.104 ***	−0.086 ***	−0.054 **	−0.090 ***	−0.082 ***	−0.062 ***
	(0.024)	(0.024)	(0.022)	(0.019)	(0.021)	(0.018)
Provincial dummies	yes	yes	yes	yes	yes	yes
Observation	10,193	10,193	10,193	10,193	10,193	10,193

Note: Standard errors are reported in parentheses. Standard errors are clustered at the municipal level. * *p* < 0.1; ** *p* < 0.05; *** *p* < 0.01.

**Table 4:** OLS estimates with endogenous independent variable lag term.

	Panel A: Lag the Viable of DIF Index by One Period					
	Nothing Could Cheer You up	Nervous	Restless and Cannot Remain Calm	Hopeless	Everything Is Difficult	Meaningless
	(1)	(2)	(3)	(4)	(5)	(6)
Digital inclusive finance	0.006 ***	0.006 ***	0.003 ***	0.005 ***	0.005 ***	0.004 ***
	(0.001)	(0.001)	(0.001)	(0.001)	(0.001)	(0.001)
Control variables	yes	yes	yes	yes	yes	yes
Provincial dummies	yes	yes	yes	yes	yes	yes
Observation	10,193	10,193	10,193	10,193	10,193	10,193
	**Panel B: Lag the Viable of DIF Index by Two Period**					
	**(7)**	**(8)**	**(9)**	**(10)**	**(11)**	**(12)**
Digital inclusive finance	0.006 ***	0.006 ***	0.002 **	0.005 ***	0.005 ***	0.004 ***
	(0.001)	(0.001)	(0.001)	(0.001)	(0.001)	(0.001)
Control variables	yes	yes	yes	yes	yes	yes
Provincial dummies	yes	yes	yes	yes	yes	yes
Observation	10,193	10,193	10,193	10,193	10,193	10,193

Note: Standard errors are reported in parentheses. Standard errors are clustered at the municipal level. ** *p* < 0.05; *** *p* < 0.01.

**Table 5:** 2SLS estimates for the impact of DIF on individual’s mental health.

	Digital Inclusive Finance	Nothing Could Cheer You Up	Nervous	Restless and Cannot Remain Calm	Hopeless	Everything Is Difficult	Meaningless
	(1)	(2)	(3)	(4)	(5)	(6)	(7)
Distance from the provincial capital	−0.113 *** (0.002)						
Digital inclusive finance		1.521 *** (0.207)	1.343 *** (0.190)	1.176 *** (0.179)	1.017 *** (0.148)	1.340 *** (0.174)	0.927 *** (0.141)
Control variables	yes	yes	yes	yes	yes	yes	yes
Provincial dummies	yes	yes	yes	yes	yes	yes	yes
Observation	10,103	10,103	10,103	10,103	10,103	10,103	10,103
F-statistic	3849.44						
	<16.38>						
LM-statistic	1921.71						
	[0.000]						

Note: Standard errors are reported in parentheses. Standard errors are clustered at the municipal level. *** *p* < 0.01.

**Table 6:** Robustness check: alternative independent variable.

	Nothing Could Cheer You Up	Nervous	Restless and Cannot Remain Calm	Hopeless	Everything Is Difficult	Meaningless
	(1)	(2)	(3)	(4)	(5)	(6)
Digital inclusive finance	0.003 ***	0.003 ***	0.002 ***	0.002 ***	0.002 ***	0.002 ***
	(0.001)	(0.000)	(0.000)	(0.000)	(0.000)	(0.000)
Control variables	yes	yes	yes	yes	yes	yes
Provincial dummies	yes	yes	yes	yes	yes	yes
Observation	10,193	10,193	10,193	10,193	10,193	10,193

Note: Standard errors are reported in parentheses. Standard errors are clustered at the municipal level. *** *p* < 0.01.

**Table 7:** Robustness check: alternative outcome variables and approaches of estimation.

	Panel A: Alternative Outcome Variables					
	Nothing Could Cheer You Up	Nervous	Restless and Cannot Remain Calm	Hopeless	Everything Is Difficult	Meaningless
	(1)	(2)	(3)	(4)	(5)	(6)
Digital inclusive finance	0.284 ***	0.195 ***	0.179 ***	0.246 ***	0.228 ***	0.254 ***
	(0.060)	(0.059)	(0.057)	(0.047)	(0.058)	(0.046)
Control variables	yes	yes	yes	yes	yes	yes
Provincial dummies	yes	yes	yes	yes	yes	yes
Observation	10,193	10,193	10,193	10,193	10,193	10,193
	**Panel B: Alternative Approaches of Estimation**					
	**(7)**	**(8)**	**(9)**	**(10)**	**(11)**	**(12)**
Digital inclusive finance	0.284 ***	0.195 ***	0.180 ***	0.246 ***	0.228 ***	0.254 ***
	(0.060)	(0.059)	(0.057)	(0.047)	(0.058)	(0.046)
Control variables	yes	yes	yes	yes	yes	yes
Provincial dummies	yes	yes	yes	yes	yes	yes
Observation	10,193	10,193	10,193	10,193	10,193	10,193

Note: Standard errors are reported in parentheses. Standard errors are clustered at the province/municipal level. *** *p* < 0.01.

**Table 8:** Mechanism exploration.

	Net Income Per Capita	Nothing Could Cheer You Up	Nervous	Restless and Cannot Remain Calm	Hopeless	Everything Is Difficult	Meaningless
	(1)	(2)	(3)	(4)	(5)	(6)	(7)
Digital inclusive finance	0.812 ***	0.478 ***	0.484 ***	0.235 **	0.415 ***	0.424 ***	0.369 ***
	(0.102)	(0.105)	(0.104)	(0.094)	(0.077)	(0.090)	(0.076)
Net income per capita		0.057 ***	0.036 ***	0.053 ***	0.044 ***	0.061 ***	0.045 ***
		(0.012)	(0.011)	(0.010)	(0.008)	(0.010)	(0.008)
Control variables	yes	yes	yes	yes	yes	yes	yes
Provincial dummies	yes	yes	yes	yes	yes	yes	yes
Observation	10,193	10,193	10,193	10,193	10,193	10,193	10,193

Note: Standard errors are reported in parentheses. Standard errors are clustered at the municipal level. ** *p* < 0.05; *** *p* < 0.01.

**Table 9:** The effect of DIF on mental health: heterogeneous effects.

	Panel A: Different Sub-Dimensions of DIF					
	Nothing Could Cheer You up	Nervous	Restless and Cannot Remain Calm	Hopeless	Everything Is Difficult	Meaningless
	(1)	(2)	(3)	(4)	(5)	(6)
Coverage breadth	−0.171 ***	−0.150 ***	−0.084 *	−0.129 ***	−0.113 **	−0.123 ***
	(0.053)	(0.051)	(0.045)	(0.038)	(0.045)	(0.035)
Use depth	0.412 ***	0.160 **	0.296 ***	0.267 ***	0.298 ***	0.215 ***
	(0.074)	(0.075)	(0.065)	(0.057)	(0.066)	(0.056)
Degree of digital service provision	−0.130 **	0.119 **	−0.090 *	−0.055	−0.021	0.031
	(0.059)	(0.059)	(0.052)	(0.043)	(0.052)	(0.042)
Control variables	yes	yes	yes	yes	yes	yes
Provincial dummies	yes	yes	yes	yes	yes	yes
Observation	10,193	10,193	10,193	10,193	10,193	10,193
	**Panel B: Subsamples by Education Level**					
	**(7)**	**(8)**	**(9)**	**(10)**	**(11)**	**(12)**
DIF	0.539 ***	0.539 ***	0.297 ***	0.477 ***	0.492 ***	0.412 ***
	(0.106)	(0.104)	(0.095)	(0.078)	(0.090)	(0.077)
Education	0.009 **	0.009 **	0.018 ***	0.015 ***	0.016 ***	0.013 ***
	(0.004)	(0.004)	(0.003)	(0.003)	(0.004)	(0.003)
DIF×Education	−0.016	−0.051 ***	−0.032 *	−0.036 **	−0.030 *	−0.011
	(0.020)	(0.019)	(0.017)	(0.015)	(0.017)	(0.014)
Control variables	yes	yes	yes	yes	yes	yes
Provincial dummies	yes	yes	yes	yes	yes	yes
Observation	10,193	10,193	10,193	10,193	10,193	10,193
	**Panel C: Subsamples by Gender**					
	**(13)**	**(14)**	**(15)**	**(16)**	**(17)**	**(18)**
DIF	0.531 ***	0.597 ***	0.274 ***	0.475 ***	0.497 ***	0.460 ***
	(0.116)	(0.114)	(0.104)	(0.086)	(0.099)	(0.087)
Male	0.130	0.300 ***	0.100	0.119	0.098	0.229 ***
	(0.101)	(0.098)	(0.088)	(0.079)	(0.088)	(0.074)
DIF×Male	−0.002	−0.143 **	0.007	−0.031	−0.037	−0.090*
	(0.071)	(0.068)	(0.061)	(0.055)	(0.061)	(0.050)
Control variables	yes	yes	yes	yes	yes	yes
Provincial dummies	yes	yes	yes	yes	yes	yes
Observation	10,193	10,193	10,193	10,193	10,193	10,193

Note: Standard errors are reported in parentheses. Standard errors are clustered at the municipal level. * *p* < 0.1; ** *p* < 0.05; *** *p* < 0.01.

## Data Availability

The data are available on request from the corresponding author.
